# Adherence to standard precautions in university hospitals during the COVID-19 pandemic: a mixed study^*^

**DOI:** 10.1590/1980-220X-REEUSP-2023-0289en

**Published:** 2024-07-15

**Authors:** Quézia Boeira da Cunha, Etiane de Oliveira Freitas, Daiane Dal Pai, José Luís Guedes dos Santos, Rosângela Marion da Silva, Silviamar Camponogara

**Affiliations:** 1 Universidade Federal de Santa Maria, Santa Maria, RS, Brazil.; 2 Universidade Federal de Santa Maria, Departamento de Enfermagem, Santa Maria, RS, Brazil.; 3 Universidade Federal do Rio Grande do Sul, Escola de Enfermagem, Porto Alegre, RS, Brazil.; 4 Universidade Federal de Santa Catarina, Departamento de Enfermagem, Florianópolis, SC, Brazil.

**Keywords:** Occupational Health, Health Personnel, Personal Protective Equipment, Precautionary Principle, COVID-19, Salud Laboral, Personal de Salud, Equipo de Protección Personal, Principio de la Precaución, COVID-19

## Abstract

**Objective:**

To analyze adherence to standard precautions by healthcare professionals and associated factors during the COVID-19 pandemic in Brazilian university hospitals.

**Method:**

Multicenter study, with a mixed approach, with a concomitant incorporated strategy and a sample of 559 health professionals and 53 managers from five university hospitals in Southern Brazil. Data collected online from September 2020 to October 2021 with the Instrument of Variables Related to Standard Precautions and sociodemographic and pandemic-related variables. Descriptive and inferential statistical analysis (Mann-Whitney and Kruskal-Wallis test) and content analysis were performed.

**Results:**

High level of adherence to standard precautions, with a significant association with having children (p = 0.014); COVID area (p < 0.001), biosafety training (p = 0.018), and social distancing (p < 0.001). The testimonies demonstrated a high risk perception and search for the use of protective equipment and biosafety knowledge.

**Conclusion:**

High adherence to standard precautions, associated with having children, working in COVID-19 care units, receiving biosafety guidance/training at the institution and practicing social distancing.

## RESUMEN

**Objetivo:**

Analizar la adherencia a las precauciones estándar por parte de los profesionales de la salud y los factores asociados durante la pandemia de COVID-19 en hospitales universitarios brasileños.

**Método:**

Estudio multicéntrico, con enfoque mixto, estrategia incorporada concomitante y muestra de 559 profesionales de la salud y 53 gestores de cinco hospitales universitarios del sur de Brasil. Datos recopilados en línea de septiembre de 2020 a octubre de 2021 con el Instrumento de Variables Relacionadas con Precauciones Estándar y variables sociodemográficas y relacionadas con la pandemia. Se realizaron análisis estadísticos descriptivos e inferenciales (prueba de Mann-Whitney y Kruskal-Wallis) y análisis de contenido.

**Resultados:**

Alto nivel de adherencia a las precauciones estándar, con asociación significativa con tener hijos (p = 0,014); área COVID (p < 0,001), capacitación en bioseguridad (p = 0,018) y distanciamiento social (p < 0,001). Los testimonios indican una alta percepción de riesgo y búsqueda del uso de equipos de protección y conocimientos de bioseguridad.

**Conclusión:**

Alto cumplimiento de las precauciones estándar, asociadas con tener hijos, trabajar en unidades de atención de COVID-19, recibir orientación/capacitación en bioseguridad en la institución y practicar el distanciamiento social.

## INTRODUCTION

The COVID-19 pandemic has exposed healthcare professionals to an increased risk of infection with the SARS-CoV-2 virus. Infection prevention and control actions were recommended to avoid and reduce as much as possible healthcare-related transmission^(1)^. These actions include the use of standard precautions (SP): measures for hygiene, infection control, and prevention of occupational exposures. The SP must be implemented in all care, regardless of patient diagnosis, considering the risk of exposure to blood or other fluids and bodily secretions^(2)^.

In the last decade, researchers have attempted to evaluate strategies to promote SP adherence among health professionals^(3)^. With the changes imposed on services following the emergence of COVID-19, new studies are being developed in order to understand SP adherence in this new context. In Brazil, authors identified positive changes in behavior regarding SP during the pandemic^(4)^. In China, researchers identified that the level of adherence to SP was positively associated with satisfaction regarding the infection control and prevention policy implemented during this period^(5)^. However, research on this issue in the context of the pandemic is still incipient.

This investigation is characterized by the integration of quantitative and qualitative results to explore SP adherence and the factors that interfered with this practice during the pandemic. The incorporation of mixed data into the analysis contributes to a deeper interpretation, providing for a comprehensive analysis of this phenomenon. Therefore, the objective of this study was to analyze adherence to standard precautions by health professionals and associated factors during the COVID-19 pandemic in Brazilian university hospitals.

## METHOD

### Study Design

Multicenter study with a concomitant embedded mixed design QUANT (qual). The quantitative study was cross-sectional and the qualitative study was exploratory-descriptive. To ensure methodological rigor, the instrument *Mixed Methods Appraisal Tool* (MMAT) was employed^(6)^.

### Local

The study scenario consisted of five large university hospitals (151 to 500 beds). These reference hospitals for COVID-19 treatment are located in five municipalities in the South region of Brazil (Curitiba – PR, Florianópolis – SC, Porto Alegre – RS, Santa Maria – RS and Rio Grande – RS). Four of them are linked to the Brazilian Hospital Services Company (*Empresa Brasileira de Serviços Hospitalares* – EBSERH).

### Population and Selection Criteria

In the quantitative study, the population was composed of nursing professionals (nurses, technicians, and nursing assistants) and physicians providing direct care to patients at least since February 2020 (when the epidemic began in Brazil). Professionals from other categories in the health sector were excluded due to the quantitative data collection instrument being aimed at care activities of physicians and nursing professionals and has been validated only for this population, avoiding, thus, a bias in data collection. During data collection, the total number of nursing and medical professionals was 10.491.

In the qualitative study, in addition to care professionals, health service managers were included, such as heads/coordinators of units and infection control, worker health, and permanent education professionals employed for at least 3 months. The inclusion of managers and other professionals aimed at a deeper understanding of the phenomenon to ensure a sufficient and reliable sample in qualitative analysis, considering that the study object is related to the actions and perceptions of all these professional groups.

### Sample Definition

The sample was selected by convenience and participants were contacted through the email address registered in their institutions. This strategy was adopted due to restrictions on contact and access to hospitals imposed by the pandemic. For quantitative collection, a sample was estimated to guarantee a significant number of participants, with a 95% confidence level and a 5% margin of error, resulting in a minimum sample of 371 health professionals.

The quantitative sample comprised 559 professionals. The qualitative sample was composed of care professionals who answered the open-ended questions at the end of the data collection instrument, amounting to 546 health professionals (nursing and physicians), in addition to 53 managers and infection control, occupational health, and permanent education professionals.

### Data Collection

The data was collected online through an electronic form on *Google Forms* from September 2020 to October 2021.

The participants provided sociodemographic data (age, sex, marital status, children) and professional data (institution, sector, professional category, employment relationship, training, predominant work shift, and length of professional experience in years).

The data collection instrument applied to obtain quantitative data was composed of two parts. The first part comprised the scales of the Instrument of Variables Related to SP, adapted and validated for use in Brazil^(7)^. This instrument is originally composed of 10 scales, seven of which were employed in this study: Adherence to SP (13 items), Prevention Effectiveness (3 items), Risk Perception (3 items), Obstacles to Following SP (6 items), Workload (3 items), Safety Climate (12 items), and Availability of PPE (2 items). These are Likert type scales with scores ranging from 1 (always/totally agree) to 5 (never/totally disagree). Summing the items, a mean was calculated; its score varies between 1 and 5 for each scale. Values lower than or equal to 3.49 meant low levels; between 3.5 and 4.49, intermediate levels; and values above 4.5 imply high levels^(7)^.

Some items were adapted so that the term “HIV” was replaced by “COVID-19”, with authorization from the author of the instrument, emphasizing the context of the pandemic. The scales were evaluated by nine judges with experience in worker health research for content validation. The judges provided their opinion on the suitability of each item that made up the scale, based on the criteria of clarity, precision, and relevance. The Content Validity Index (CVI) obtained for each item was ≥ 0.80, attesting to the validity of the instrument^(8)^.

The second part of the quantitative data instrument was a questionnaire containing 11 closed questions, which addressed issues related to the pandemic, such as: receiving biosafety guidance from the institution with a focus on the transmission of the new coronavirus; complying with the recommendation of social distancing in private life activities; having experienced COVID-19 symptoms; using PPE during direct care to suspected or confirmed COVID-19 patients; having been tested; being part of a risk group, among others.

A questionnaire with open questions about protective measures during the pandemic was employed for qualitative data collection. The instrument was composed of five open questions focusing on issues related to: perception of use of individual protection measures by the workers; difficulties and strategies to improve protection in the workplace; perception of the institution’s action on this issue. The questionnaire was incorporated into the quantitative research protocol; however, its completion was not mandatory.

### Data Analysis and Treatment

The quantitative data were organized in an electronic spreadsheet in the form of a database in Excel/Windows and were analyzed in IBM-SPSS version 25. Data analysis was performed using descriptive statistics (absolute and relative frequency) and inferential statistics (Mann-Whitney or Kruskal-Wallis test). The Shapiro-Wilk normality test showed that the variables did not present a normal distribution. The scales of the Instrument of Variables Related to SP were represented by the median and interquartile range, taking into account the distribution of the data. A 5% (p < 0.05) significance level was adopted for all analyses.

The qualitative data were submitted to content analysis^(9)^ with the aid of MAXQDA software. After organizing the data set to be analyzed (1st stage), exploration and in-depth analysis of the material was carried out, with coding and grouping (2nd stage). At this stage, the data was grouped into a category with 13 subcategories. Finally, the results were processed and interpreted (3rd stage).

The qualitative data were then incorporated into quantitative data based on the comparison of findings, so that items from the qualitative category were incorporated into the quantitative results. This integration was carried out during result interpretation to complement the information and provide a deeper understanding of the study object.

### Ethical Aspects

This study was submitted to the Research Ethics Committee of the five participating institutions and was approved under the following opinions: n. 4,335,006, n. 4,466,661, n. 4,685,755, n. 4,348,898, and n. 4,501,805. All ethical principles foreseen in research involving human beings were respected in accordance with Resolution 466/12. Study participation was preceded by online participant consent through the Informed Consent Form. Furthermore, to preserve anonymity, qualitative responses were identified by codes composed of the letter “E” for nurses, “M” for physicians, “TE” for nursing technicians, “AE” for nursing assistants, and “G” for managers and infection control, worker health, and permanent education professionals, followed by numbers associated with the order in which the participant was integrated into the study. The institutions were also identified by letters: Hospital A, Hospital B, and so on.

## RESULTS

A total of 559 professionals participated in the quantitative analysis, 71% (n = 397) of which were nursing professionals (195 nursing technicians nursing, 185 nurses, 17 nursing assistants). In this sample, 77.3% (n = 432) were female, with a mean age of 45 (±9.54) years (minimum 22 and maximum 68 years old); 79.4% (n = 444) had a partner, and 72.8% (n = 407) had children. Regarding the professional variables, 35.4% (n = 198) were Specialists and 72.4% (n = 405) had their employment contract governed by the Consolidation of Labor Laws (*Consolidação das Leis do Trabalho* – CLT).

The average length of professional experience was 18.3 years (SD ± 9.1) and the predominant work shift at the institution was daytime for 67.1% (n = 375). A higher percentage (77.6%, n = 434) reported having an employment relationship and 82.3% (n = 460) had a weekly workload of ≤ 40 hours.

Table 1 provides data on the association between sociodemographic and occupational characteristics and adherence to SP.

**Table 1:** Comparison of the distribution of adherence to standard precautions among sociodemographic and occupational categorical variables (n = 559). Curitiba – Paraná; Florianópolis – Santa Catarina; Porto Alegre, Santa Maria e Rio Grande – Rio Grande do Sul, Brazil, 2020–2021.

n (%)	Adherence to SP med [P25; P75]	p-value
**Sex**		
Female 432 (77.3)	4.69 [4.46; 4.85]	0.440*
Male 127 (22.7)	4.62 [4.31; 4.85]	
**Marital status**		
Partnered 444 (79.4)	4.69 [4.46; 4.85]	0.943*
No partner 115 (20.2)	4.69 [4.38; 4.85]	
**Children**		
Yes 407 (72.8)	4.69 [4.46; 4.85]	0.014*
No 152 (27.2)	4.62 [4.38; 4.85]	
**Sector**		
COVID area 53 (9.5)	4.77 [4.69; 4.92]	<0.001*
Non-COVID area 506 (90.5)	4.69 [4.38; 4.85]	
**Professional category**		
Nursing 397 (71)	4.69 [4.46; 4.85]	0.630*
Physicians 162 (29)	4.69 [4.38; 4.85]	
**Employment relationship**		
RJU 127 (22.7)	4.69 [4.46; 4.85]	0.132**
CLT 405 (72.5)	4.69 [4.38; 4.85]	
Emergency 27 (4.8)	4.77 [4.62; 4.92]	
**Education (concluded)**		
Secondary 118 (21.1)	4.69 [4.54; 4.85]	0.347**
Tertiary 71 (12.7)	4.69 [4.38; 4.92]	
Specialist/resident 198 (35.4)	4.69 [4.38; 4.85]	
Master’s 117 (21.0)	4.62 [4.38; 4.77]	
PhD 54 (9.7)	4.69 [4.46; 4.92]	
**Predominant work shift**		
Daytime 375 (67.1)	4.69 [4.38; 4.85]	0.582*
Night 183 (32.7)	4.69 [4.46; 4.85]	
**Other employment relationship**		
No 434 (77.6)	4.69 [4.46; 4.85]	0.999*
Yes 125 (22.4)	4.69 [4.38; 4.85]	
**Weekly workload**		
≤40h 460 (82.3)	4.69 [4.46; 4.85]	0.921*
>40h 99 (17.7)	4.69 [4.38; 4.85]	

*Mann-Whitney test; **Kruskal-Wallis test. SP: standard precaution; RJU: Single Legal Regime; CLT: Consolidation of Labor Laws.

The medians of the scales of the SP-Related Variables Instrument are described in Table 2. A high level was found for “adherence to SP” and intermediate levels for the other variables.

**Table 2:** Median and interquartile range of variables related to standard precautions (n = 559). Curitiba – Paraná; Florianópolis – Santa Catarina; Porto Alegre, Santa Maria e Rio Grande – Rio Grande do Sul, Brazil, 2020–2021.

Scale	med [P25; P75]
Adherence to SP	4.69 [4.38; 4.85]
Prevention effectiveness	4.00 [3.66; 4.67]
Risk perception	4.00 [3.33; 4.67]
Obstacles to following SP	4.00 [3.50; 4.67]
Workload	4.00 [3.67; 4.67]
Safety climate	3.83 [3.25; 4.33]
PPE availability	4.00 [3.50; 5.00]

SP: standard precaution; PPE: personal protective equipment.

The pandemic-related variables associated to higher values of SP adherence were described in Table 3. Professionals who received biosafety guidance and/or training at the institution with a focus on preventing coronavirus transmission had significantly higher values of SP adherence. Those who stated always complying with social distancing recommended by the World Health Organization (WHO) in other private life activities also had significantly higher values.

**Table 3:** Comparison between medians of adherence to standard precautions and COVID-19 pandemic variables (n = 559). Curitiba – Paraná; Florianópolis – Santa Catarina; Porto Alegre, Santa Maria e Rio Grande – Rio Grande do Sul, Brazil, 2020-2021.

n (%)	Adherence to SP med [P25; P75]	p-value
**Biosafety guidance and/or training at the institution with a focus on preventing the transmission of the new coronavirus**		
Yes 523 (93.6)	4.69 [4.46; 4.85]	0.018*
No 36 (6.4)	4.50 [4.15; 4.77]
**Have you complied with social distancing as recommended by the WHO in other activities in your private life?**		
Always 277 (49.6)	4.69a [4.54; 4.92]	<0.001**
Most of the time 259 (46.3)	4.62b [4.38; 4.85]	
Occasionally 13 (2.3)	4.46ab [4.23; 4.69]	
Rarely 10 (1.8)	4.58ab [4.00; 4.85]	

*Mann-Whitney test; **Kruskal-Wallis test; Dunn’s post-hoc: different letters represent statistically different distributions. SP: standard precaution; WHO: World Health Organization.

The significant associations between the SP variables and pandemic variables are described in Table 4. The median risk perception was higher in the group of participants who had symptoms suggestive of COVID-19 during the period. The group that affirmed having receiving biosafety guidance/training with a focus on preventing the transmission of the new coronavirus had a better perception regarding the safety climate and a better perception regarding the availability of PPE. The group that affirmed always using PPE during direct care to patients with a suspicion or confirmation of COVID-19 infection had a better perception of the safety climate and the availability of PPE.

**Table 4:** Comparison of the medians of the Instrument of Variables Related to Standard Precautions with the variables related to the COVID-19 pandemic (n = 559). Curitiba – Paraná; Florianópolis – Santa Catarina; Porto Alegre, Santa Maria e Rio Grande – Rio Grande do Sul, Brazil, 2020-2021.

n(%)	Risk perception med [P25; P75]	p-value	Safety climate med [P25; P75]	p-value	PPE availability med [P25; P75]	p-value
**Symptoms suggestive of COVID-19**						
Yes 224 (40.1)	4.33 [3.67; 4.67]	<0.001*	3.83 [3.17; 4.25]	0.218*	4.00 [3.50; 5.00]	0.118*
No 335 (59.9)	4.00 [3.33; 4.33]		3.83 [3.33; 4.33]		4.50 [4.00; 5.00]	
**Biosafety guidance and/or training at the institution with a focus on preventing the transmission of the new coronavirus**						
Yes 523 (93.6)	4.00 [3.33; 4.67]	0.812*	3.92 [3.33;4.33]	<0.001*	4.50 [4.00; 5.00]	0.001*
No 36 (6.4)	4.00 [3.33; 4.67]		3.21 [2.67; 3.50]		3.50 [2.75; 4.50]	
**Use of PPE when providing care and/or performing procedures to a patient suspected or confirmed for COVID-19**						
Always 460 (82.3)	4.00 [3.33; 4.67]	0.169**	3.92a [3.33; 4.33]	0.001**	4.50a [4.00; 5.00]	0.001**
Most of the time 92 (16.5)	4.00 [3.33; 4.67]		3.33b [2.96; 3.92]		3.50b [2.50; 4.00]	
Occasionally 4 (0.7)	3.50 [3.17; 4.00]		2.58ab[1.75; 3.54]		2.75b [1.75; 3.00]	
Rarely 3 (0.5)	4.67 [4.33; 5.00]		2.83a [2.67; 4.17]		3.50a [3.50; 4.00]	

*Mann-Whitney test; **Kruskal-Wallis test; Dunn’s post-hoc: different letters represent statistically different distributions. PPE: personal protective equipment.

The findings of the qualitative analysis were grouped into a category entitled “Repercussions of the pandemic on SP adherence”, which demonstrated the professionals’ understanding of protective measures, the perception of risks, and issues related to the work environment and institutions during this period. The unit of analysis used to construct the category was “Factors related to SP adherence”. This category was presented in 13 subcategories, which were incorporated into the quantitative results. Significant statements were extracted to better represent them, as described in the Joint display shown in Chart 1.

**Chart 1:** Joint display integrating quantitative results and participant statements. Curitiba – Paraná; Florianópolis – Santa Catarina; Porto Alegre, Santa Maria e Rio Grande – Rio Grande do Sul, Brazil, 2020-2021.

Repercussions of the pandemic on adherence to standard precautions		
Subcategory	Quantitative results (n = 559)*	Qualitative results (n = 546 + 53)**
Adherence to SP	Adherence to PP [med] = 4.69 67% of participants stated that they follow PP with all patients, regardless of diagnosis.	*“The implementation of standard precautions constituted the main measure to prevent the transmission of COVID-19 among patients and healthcare professionals and was adopted in care to all patients, minimizing exposure to respiratory pathogens, including COVID-19.”* G46, Hospital C – Head of nursing at the Surgical Center “*I think that the pandemic improved our reflection on the importance of standard precautions in our daily work.”* G6, Hospital D – Head of the Surgery Unit and Material and Sterilization Center
Use of PPE	Apron: 66% always use it when there is a possibility of getting blood or other secretions on their clothes. Gloves: 88% always use them when there is a possibility of contact with blood or other secretions. Protective glasses or face shield: 50% always use them when there is a possibility of blood or other secretions splashing into the eyes. Mask: 89% always use it when there is a possibility of blood or other secretions splashing into the mouth.	*“The workers, faced with the pandemic, understood the need of using PPE to protect themselves. Obvious protections were not taken seriously during daily activities, by some, before the pandemic”*. G32, Hospital B – Occupational Health and Safety Service *“Adherence to the use of PPE is based on a good job at raising awareness. This is now being done. Healthcare professionals are finally wearing PPE without us having to beg for it.”* G53, Hospital D – Risk Management Unit “*I have adopted PPE more consciously*.” TE48, Hospital A – ICU *“The upside to all this was and is daily learning that came with the pandemic and the reinforcement of existing practices, with a more conscious adoption. For example, PPE. ”* E133, Hospital C – Psychiatry
Hand hygiene	75% always washed their hands after removing disposable gloves.	*“The professionals learned to value precautions to protect themselves and patients, trying to prevent outbreaks and, above all, they learned to use PPE. There has never been so much talk about hand hygiene.”* G13, Hospital A - Hospital Infection Control Service
Care for materials and the environment	86% considered all materials in contact with saliva from patients to be contaminated.	*“My work environment serves COVID and non-COVID patients, and it is very important for the environment not to be contaminated. We keep an eye on everyone who leaves the room and does not care for the environment.”* E123, Hospital C – Hemodynamics
Family	Significant association between adherence to SP and “having children” (p = 0.014).	*“The frightening possibility of contracting it and transmitting it to other people, whether patients, family, and friends, has shaped many attitudes. Daily practices regarding the use of PPE were widely followed.”* E156, Hospital A – COVID UNIT
Work in the COVID area	Greater adherence to SP in the COVID area (p < 0.001). Professionals who worked in the “COVID area” had a perception of fewer “obstacles to following SP” and a better assessment of the “safety climate” and “PPE availability”.	*“It is a place with a greater probability of contracting the disease, but safer in terms of protection, as we protect ourselves more with PPE.”* TE15, Hospital A – COVID UNIT “*In the COVID area, employees dress appropriately. However, in the non-COVID area, there is some relaxation in the use of precautionary measures*.” G28, Hospital C – Nursing Education Service *“... compliance within the COVID ICU is visibly greater considering the professional’s protection bias.”* G9 – Hospital D – Head of the Intensive Care Unit
Perception of protection effectiveness	86% of professionals believe they can reduce the risk of acquiring COVID-19 at work if they follow standard precautions.	*“I believe that healthcare professionals wearing everything correctly have a reduced risk of acquiring the virus at work, we definitely take better care of ourselves.”* M92, Hospital B – Pediatrics
Risk perception	6% of participants do not feel exposed to contracting COVID-19 at work. “Risk perception” is higher among nursing professionals (p < 0.001) and in the group of participants who had symptoms suggestive of COVID-19 (p < 0.001).	*“The pandemic caused fear among professionals, leading many to use PPE appropriately. However, there are moments of denial, in which professionals ignore the indicated protection measures.”* G27, Hospital C – Head of the Psychiatric Nursing Service *“It is difficult to understand why some people do not want to accept that the virus is lethal and often do not follow the protection standards.”* TE111, Hospital B – Outpatient Unit
Obstacles to SP adherence	24% stated that the accumulation of daily activities frequently interferes with their ability to follow the SP. Around 20% stated that they cannot get used to using PPE when carrying out some tasks and believe that following the SP makes work more difficult.	*“Face shield distorts the vision and interferes with tracheal intubation.”* M47, Hospital C – Surgical Center *“Pressure for agility in service that is not always possible having to follow clothing protocols.”* TE48, Hospital A – ICU *“Sometimes it is difficult to equip myself quickly when an emergency that requires speed arises.”* M118, Hospital D – Emergency Room *“It is very difficult to hear each other with a mask and face shield. We have to scream and that makes the work much more exhausting.”* M48, Hospital C – ICU
Workload	A total of 35% of the participants stated that there is always much work to be done. Physicians had a greater perception of “workload” compared to nursing professionals (p = 0.002).	*“In relation specifically to the hospital, having to perform activities that in other institutions are nursing responsibilities (such as collecting nasopharyngeal swab, ECG, measuring vital signs of patients with respiratory symptoms), which makes work even more exhausting.”* M4, Hospital D – Emergency Room
Safety climate	Safety climate was the scale with lowest score (Md = 3.83). 68% completely agree that they have support from their supervisor to follow the SP. 16% completely agree that in their institution senior management is personally involved in security activities. Hospital C had the best “safety climate” assessment (4.17) and Hospital D had the worst (3.25) (p < 0.001). Physicians (p = 0.009) and CLT professionals (p < 0.001) had a better assessment of this item.	*“A bit flawed on the part of managers, who DO NOT work in care and have a slightly distorted perception of work routines.”* TE80, Hospital D – ICU “*I realize that the institution just wants ‘labor’, it is not truly concerned with the employee’s (physical and mental) health*.” TE20, Hospital D – COVID UNIT *“I feel the Hospital is very committed to the safety of all its professionals. It meets all our protective needs.”* E102, Hospital C - General Ward *“Since the pandemic started, we all received orientation from the management. And this made us very confident to deal with all situations in the face of the pandemic.”* TE84, Hospital C - ICU *“Effective, correct guidelines. The managers collaborated with the team, questioning what the best evidence for team safety was.”* TE146, Hospital C – COVID UNIT *“When the pandemic began, support from the institution was focused on intensive care units, as if it were the only means of transmission. I did not feel safe. Afterwards, with the emergence of cases and contamination in the units, the processes improved.”* E133, Hospital C – Psychiatry
PPE availability	48% of all participants stated that their work unit has all the necessary equipment and material to protect against COVID-19. Physicians (p = 0.04) had a more positive perception of “PPE availability”.	“...*when the pandemic began, the correct number of PPE was not available for the entire team and N95 masks were initially being dispensed only for medical, physiotherapy, and nursing teams, and not for nursing technicians, which caused discomfort*...” E2, Hospital A – Surgical Center *“I think everything possible was done. Sometimes we have PPE of questionable quality, such as disposable aprons, but there was never a lack of material, which I consider an achievement worth highlighting.”* M91, Hospital B – ICU
Biosafety training/qualification	Having received biosafety guidance/training with a focus on preventing transmission of the new coronavirus was associated with higher levels of adherence to SP (p = 0.018).	*“There was greater concern for obtaining knowledge and searching for suitable material.”* G11, Hospital D – Hospital Infection Control Service *“What we have in a positive way are enlightening live streams regarding the subject.”* E16, Hospital A – Outpatient Ward

*n = 559: nursing professionals and physicians who worked in care. **n = 543 + 53: nursing professionals and physicians in care + health service managers and professionals in infection control, worker health, and permanent education services.

## DISCUSSION

The results made it possible to analyze adherence to SP by health professionals during the pandemic. Previous studies presented intermediate levels of adherence to SP^(10–13)^, different from the high level found in this study. The qualitative responses corroborate this finding, demonstrating that the pandemic led to greater awareness regarding the use of SP.

The fear of contracting and transmitting the disease to family members was identified as an aspect that influenced adherence to SP, whereas professionals who have children were more adherent. The context of the pandemic made professionals reflect on the risks to which they are exposed in the work environment, generating changes in attitudes and behaviors, which resulted in greater adherence to SP. The literature points out that the fear of becoming infected or infecting family members is among the main mental challenges faced by health professionals who work in pandemics, which, added to the stigma from society, generates stress and isolation^(14)^. Thus, adhering to the SP, in addition to the benefit of physical protection, contributes to the emotional well-being of workers, who then feel safe to perform their tasks.

The highest adherence to SP was observed among professionals working in units exclusively dedicated to the treatment of COVID-19 patients. According to the qualitative results, the professionals in these units had less difficulty in following the SP and received more institutional support, both regarding the availability of equipment and the active participation of managers alongside the teams. Added to this, the high risk of contamination in these locations is believed to have contributed positively to the motivation for professionals to adhere to safety practices rigorously.

A high safety climate in COVID-19 units was observed in other countries. In Spain, professionals in these areas felt privileged because they were sufficiently equipped and high rates of contagion were not observed among them^(15)^. Other studies have shown that contamination among health professionals was higher in general wards, a fact that researchers attribute to a difference in the use of PPE and biosafety practices between places for exclusive care for patients with COVID-19 and places providing general care^(16, 17)^.

This study’s findings demonstrated that professionals who adhere more to the use of PPE when providing care to patients with COVID-19 had a better perception regarding the safety climate and availability of PPE, evidencing that feeling supported is a relevant factor in encouraging adherence to SP.

It is important to highlight that the safety climate perceived by the participants had a significant difference among the institutions. In the institution that had the best evaluation on this scale, Hospital C, participants reported an active participation of managers with the teams, with clear and effective guidelines, seeking the best evidence for everyone’s safety. This made professionals realize the institution’s commitment to the safety of professionals, seeking to meet their protection needs, which improved their safety climate perception.

In this sense, the safety climate item evaluated as the worst by study participants was the involvement of senior management in security activities. A study with Brazilian nursing professionals showed that the majority (81.8%) of them did not feel safe with the actions implemented by institutions in combating COVID-19^(18)^. The feeling of inadequate support, combined with a high workload and lack of PPE, has an impact on the mental burden of professionals^(19)^. Professionals need to feel supported by their institutions and leaders and, thus, encouraged to take responsibility for a safer working environment.

Health professionals demonstrated a high interest in learning about biosafety measures. In addition, health institutions offered various types of training, qualifications, and guidance on the subject, which favored the dissemination of knowledge related to SP. The variable “Having received biosafety guidance and training” was statistically associated with a better perception of the safety climate and availability of PPE. Also, the professionals who adhered more strongly to the SP were those who received training at the institution.

Regarding the availability of PPE, it was identified that a percentage of 48% stated that their work units had all the equipment necessary for their protection. In the qualitative analysis, it was identified that the units designed to provide care to patients with COVID-19 received a privileged supply of this equipment, which was also demonstrated in the literature^(15)^. A study carried out in the ICU of a university hospital in Canada found that the availability of PPE was a constant concern during the pandemic. The professionals’ anguish was related to the possibility that there would not be enough equipment, in addition to the need to use hitherto unknown products^(20)^. In this regard, it is important to emphasize that having the conditions to carry out safe work, especially in a time of health crisis, is relevant to the physical and mental health of health professionals.

Adherence to SP was associated with compliance with the social distancing recommendation. The individuals who most closely followed safety regulations in the workplace extended this caution to the extra-hospital environment, signaling coherent attitudes in the search for greater protection against virus infection. These actions are essential to be carried out together, considering that the effectiveness of social distancing depends largely on the adoption of other measures, such as correct hand hygiene, wearing masks, and surface hygiene measures^(21)^.

Understanding the risk exposure is considered an important factor for the attitude of health professionals regarding the use of protective measures. The findings of the present study demonstrated that professionals with COVID-19 symptoms had a greater risk perception for SARS-CoV-2 infection. This aspect is important because lack of awareness of risks contributes to unsafe behavior. A study demonstrated that, despite serious work accidents involving biological material, the risks are underestimated by both employers and employees^(22)^. During the pandemic, exposure to a previously unknown risk increased the awareness of professionals, influencing the adoption of protective measures. However, it is necessary to consider that permanent exposure favors the reduction of risk perception, making the decision to adopt SP^(22)^ more difficult. Thus, new studies and interventions will have to be developed in the following years to better evaluate changes in risk perception and its association with the adoption of safety measures by health professionals.

The results also showed a high adherence to PPE, such as apron, mask, gloves, and protective glasses. This result is positive when compared to a previous study carried out only with nursing professionals^(12)^. Both the quantitative and qualitative results showed that the participants believed it was possible to provide care to infected patients without contaminating themselves by employing adequate protection. In fact, studies demonstrate that compliance with the indications for the use of PPE is efficient for preventing infections among health professionals^(23)^. In China, researchers sought to examine the protective effects of PPE for professionals providing care to COVID-19 patients and demonstrated that, despite the high risk of exposure, none of the 420 professionals were infected, even when performing aerosol-generating procedures, as they were adequately protected^(24)^.

Despite the benefit of protection, the use of PPE has negative implications for work. Around 20% stated that they were unable to get used to PPE and believed that following the SP made work more difficult. The difficulty in carrying out tasks with PPE involves the reduction of senses, such as vision, hearing, and touch, interfering with the ability to carry out work efficiently. Furthermore, the physical discomfort of using PPE made adherence for prolonged periods a challenge to be overcome. A survey of nurses pointed out the most common discomforts related to the use of PPE: sweating when using a surgical mask (50.9%) or the N95 type mask (64.2%), dry hands due to the constant washing and using gloves (73.9%), sweating when wearing coveralls/aprons (84.1%), and vision problems and headaches when using protective glasses/face shields (47.9%)^(25)^. These discomforts need to be considered in increasing adherence, indicating the need for more research on PPE quality, effectiveness, and comfort.

In addition to the use of PPE, an increase in hand hygiene was one of the changes reported by the participants. However, the quantitative results pointed out that 75% of professionals washed their hands after removing gloves. Studies carried out before the pandemic demonstrated a greater practice of hand hygiene, reaching 83.6% among nursing professionals at a university hospital^(12)^ and 82.9% in another study carried out in a psychiatric hospital^(11)^.

A study carried out in Singapore verified environmental contamination in isolation areas for COVID-19 patients at distances greater than one meter, demonstrating that the contamination was probably caused by the hands of healthcare professionals^(26)^. In a sample of 72 healthcare professionals in Wuhan, China, researchers showed that unqualified handwashing, suboptimal hygiene before contact with the patient, and inadequate use of PPE were risk factors for infection by SARS-CoV-2 in workers^(27)^. It is thus clear the central role that adherence to the SP has both in the protection of health professionals and the safety of hospitalized patients, alleviating concerns so that professionals stop being vectors of infection in the hospital environment through strict application of biosafety measures.

Even though the results of this study are supported by the literature, some limitations can be highlighted. The use of a self-administered instrument, which is subject to the participant’s memory bias, may have interfered with the results. This study’s cross-sectional design makes it impossible to establish cause and effect relationships. Finally, we recognize the non-probabilistic characteristic of the sample, which had more participants from the nursing category, even though the study was disseminated also to medical professionals from the participating institutions.

The findings of this study may guide new interventions in the education process in institutions, seeking to protect both professionals and patients. The strategies must be focused on consistent changes in the risk behavior of professionals, in addition to improving working conditions, which involves adequate staffing and provision of sufficient, quality material and PPE for all professionals providing care or exposed to its risks. Support from institutions must include the participation of the entire team in security activities, from care to the management.

Monitoring adherence to safety practices is recommended, as well as the maintenance of educational strategies and the creation of institutional programs for worker health care.

## CONCLUSION

The study identified a high level of adherence to SP by healthcare professionals during the COVID-19 pandemic. The variables related to SP were found to be at intermediate levels. Factors associated with greater adherence to SP were having children, working in COVID-19 care units, receiving biosafety guidance/training at the institution, and complying with social distancing as recommended by the WHO. The high risk perception of health professionals also contributed positively to adherence to SP during this period, as well as an expressive search for use of protective equipment and the propagation of biosafety knowledge.
