# Supplementary material for: Adherence to standard precautions in university hospitals during the COVID-19 pandemic: a mixed study
Source: Rev Esc Enferm USP. 2024 Jul 15;58:e20230289. doi: 10.1590/1980-220X-REEUSP-2023-0289en (PMC11259396; doi:10.1590/1980-220X-REEUSP-2023-0289en)
Supplement: Translation

# Translation: Adesão às precauções padrão em hospitais universitários na pandemia de COVID-19: estudo misto^*^

**DOI:** 10.1590/1980-220X-REEUSP-2023-0289pt

**Authors:** Quézia Boeira da Cunha, Etiane de Oliveira Freitas, Daiane Dal Pai, José Luís Guedes dos Santos, Rosângela Marion da Silva, Silviamar Camponogara

**Affiliations:** 1 Universidade Federal de Santa Maria, Santa Maria, RS, Brasil.; 2 Universidade Federal de Santa Maria, Departamento de Enfermagem, Santa Maria, RS, Brasil.; 3 Universidade Federal do Rio Grande do Sul, Escola de Enfermagem, Porto Alegre, RS, Brasil.; 4 Universidade Federal de Santa Catarina, Departamento de Enfermagem, Florianópolis, SC, Brasil.Departamento de Enfermagem

**Keywords:** Saúde do Trabalhador, Pessoal de Saúde, Equipamento de Proteção Individual, Princípio da Precaução, COVID-19

## Abstract

**Objetivo:**

Analisar a adesão às precauções padrão por profissionais da saúde e os fatores associados durante a pandemia de COVID-19 em hospitais universitários brasileiros.

**Método:**

Estudo multicêntrico, de abordagem mista, com estratégia incorporada concomitante e amostra de 559 profissionais da saúde e 53 gestores de cinco hospitais universitários do Sul do Brasil. Coleta de dados realizada de setembro de 2020 a outubro de 2021, online, com o Instrumento de Variáveis Relativas às Precauções Padrão, variáveis sociodemográficas e relacionadas à pandemia. Realizada análise estatística descritiva e inferencial (teste de Mann-Whitney e Kruskal-Wallis) e análise de conteúdo.

**Resultados:**

Alto nível de adesão às precauções padrão, com associação significativa para ter filhos (p = 0,014); área COVID (p < 0,001), treinamento sobre biossegurança (p = 0,018) e distanciamento social (p < 0,001). Depoimentos demonstraram percepção de risco elevada e busca pela utilização de equipamentos de proteção e por conhecimentos relacionados à biossegurança.

**Conclusão:**

Alta adesão às precauções padrão, associada a ter filhos, trabalhar em unidades de atendimento à COVID-19, receber orientações/capacitação sobre biossegurança na instituição e realizar distanciamento social.

## INTRODUÇÃO

A pandemia de COVID-19 expôs os profissionais da saúde a um risco aumentado de infecção pelo vírus SARS-CoV-2. Ações de prevenção e controle de infecção foram recomendadas com o objetivo de evitar e reduzir ao máximo a transmissão do vírus durante a assistência à saúde^(1)^. Entre essas ações, ressalta-se a utilização das precauções padrão (PP), que são medidas de higiene, controle de infecção e de prevenção de exposições ocupacionais. As PP devem ser implementadas em todos os atendimentos, independentemente do diagnóstico do paciente, mediante o risco de exposição a sangue ou outros fluidos e secreções corporais^(2)^.

Na última década, pesquisadores buscaram avaliar estratégias para promover a adesão às PP entre os profissionais da saúde^(3)^. Com as mudanças impostas aos serviços a partir do surgimento da COVID-19, novas pesquisas vêm sendo desenvolvidas no sentido de compreender como foi a adesão às PP nesse novo cenário. No Brasil, autores identificaram mudanças positivas de comportamento em relação ao uso das PP durante a pandemia^(4)^. Na China, os pesquisadores identificaram que o nível de adesão às PP foi positivamente associado à satisfação com a política de controle e prevenção de infecções implementada nesse período^(5)^. Contudo, as pesquisas sobre essa problemática no contexto da pandemia ainda são incipientes.

Esta investigação tem como diferencial a integração de resultados quantitativos e qualitativos para explorar a adesão às PP e os fatores que interferiram nessa prática durante a pandemia. A incorporação de dados mistos na análise contribui para uma interpretação mais aprofundada, permitindo analisar com mais abrangência esse fenômeno. Sendo assim, o objetivo do estudo foi analisar a adesão às precauções padrão por profissionais da saúde e os fatores associados durante a pandemia de COVID-19 em hospitais universitários brasileiros.

## MÉTODO

### Tipo de Estudo

Estudo multicêntrico, com abordagem metodológica mista e estratégia incorporada concomitante QUANT (qual). O estudo quantitativo caracterizou-se por ser do tipo transversal e o estudo qualitativo teve caráter exploratório-descritivo. A fim de assegurar o rigor metodológico, foi utilizado o instrumento *Mixed Methods Appraisal Tool* (MMAT)^(6)^.

### Local

O cenário do estudo constituiu-se de cinco hospitais universitários de grande porte (de 151 a 500 leitos); todos eram referência para tratamento da COVID-19 e estão localizados em cinco municípios na região Sul do Brasil (Curitiba – PR, Florianópolis – SC, Porto Alegre – RS, Santa Maria – RS e Rio Grande – RS), sendo quatro deles vinculados à Empresa Brasileira de Serviços Hospitalares (EBSERH).

### População e Critérios de Seleção

No estudo quantitativo, a população foi composta por profissionais da enfermagem (enfermeiros, técnicos e auxiliares de enfermagem) e médicos que atuavam na assistência direta aos pacientes pelo menos desde fevereiro de 2020 (período de início da epidemia no Brasil). Foram excluídos os profissionais de outras categorias da área da saúde devido ao instrumento de coleta de dados quantitativo escolhido ser voltado para as atividades assistenciais de médicos e profissionais da enfermagem e ter sido validado apenas nessa população, evitando-se, dessa forma, um viés na coleta de dados. No período de coleta de dados, o número total de profissionais da enfermagem e médicos era de 10.491.

No estudo qualitativo, além dos profissionais da assistência, foram incluídos gestores dos serviços de saúde, como chefes/coordenadores das unidades, profissionais que atuavam nos serviços de controle de infecção, saúde do trabalhador e serviço de educação permanente havia pelo menos 3 meses. A inclusão dos gestores e demais profissionais foi realizada com a finalidade de aprofundar a compreensão do fenômeno investigado e, dessa forma, garantir uma amostra suficiente e fidedigna na análise qualitativa, tendo em vista que a dimensão do objeto investigado se articula com as ações e percepções de todos esses grupos profissionais.

### Definição da Amostra

A amostra foi selecionada por conveniência e os participantes foram recrutados pelo e-mail cadastrado junto às instituições. Essa estratégia foi adotada devido às restrições de contato e de acesso aos hospitais impostas pela situação de pandemia. Para coleta quantitativa, foi realizado um cálculo para estimativa amostral, com a finalidade de garantir um número significativo de participantes, com nível de confiança de 95% e margem de erro de 5%, resultando em uma amostra mínima de 371 profissionais de saúde.

A amostra de respondentes obtida para os dados quantitativos foi de 559 profissionais. A amostra de dados qualitativos foi composta pelos profissionais assistenciais que responderam às questões abertas ao final do instrumento de coleta de dados, os quais somaram 546 profissionais de saúde (enfermagem e médicos), além de 53 gestores e profissionais do serviço de controle de infecção, saúde ocupacional e educação permanente.

### Coleta de Dados

A coleta de dados foi realizada online, via formulário eletrônico pela plataforma *Google Forms,* no período de setembro de 2020 a outubro de 2021.

Foram coletados dados de caracterização sociodemográfica (idade, sexo, situação conjugal, filhos) e profissional (instituição, setor, categoria profissional, vínculo de trabalho, formação concluída, turno de trabalho predominante e tempo de experiência profissional em anos) de todos os participantes.

O instrumento de coleta de dados aplicado para obtenção de dados quantitativos era composto por duas partes. A primeira parte continha as escalas do Instrumento de Variáveis Relativas às PP, adaptado e validado para uso no Brasil^(7)^. Esse instrumento é originalmente composto por 10 escalas, sendo que neste estudo foram utilizadas sete: Adesão às PP (13 itens), Eficácia da Prevenção (3 itens), Percepção de Risco (3 itens), Obstáculos para Seguir às PP (6 itens), Carga de Trabalho (3 itens), Clima de Segurança (12 itens) e Disponibilidade de EPI (2 itens). As escalas desse instrumento são do tipo *likert*, cuja pontuação varia de 1 (sempre/concordo totalmente) a 5 (nunca/discordo totalmente). A partir da soma dos itens, foi feita uma média cuja pontuação varia entre 1 e 5 para cada escala. Valores menores ou iguais a 3,49 significam níveis baixos; entre 3,5 e 4,49, níveis intermediários; e valores acima de 4,5 implicam níveis altos^(7)^.

Esse instrumento passou por uma adaptação na redação de alguns itens, de forma que o termo “HIV” foi substituído por “COVID-19”, com autorização da autora do instrumento, enfatizando o contexto da pandemia. As escalas foram submetidas à avaliação de nove juízes com experiência em pesquisas na área de saúde do trabalhador para validação do conteúdo. Os juízes opinaram sobre a adequação de cada item que compunha a escala, com base nos critérios de clareza, precisão e relevância. O Índice de Validade de Conteúdo (IVC) obtido em cada item foi ≥ 0,80, atestando a validade do instrumento^(8)^.

A segunda parte do instrumento de dados quantitativos era um questionário contendo 11 questões fechadas, o qual abordava questões relacionadas à pandemia, como: receber orientações da instituição sobre biossegurança com foco na transmissão do novo coronavírus; cumprimento da recomendação de distanciamento social nas atividades da vida particular; ter tido sintomas de COVID-19; utilização de EPI durante a assistência direta a pacientes suspeitos ou confirmados de COVID-19; ter sido testado; fazer parte do grupo de risco, entre outras.

Na coleta de dados qualitativos, foi utilizado um questionário com questões abertas sobre as medidas protetivas durante a pandemia. O instrumento foi composto por cinco perguntas abertas que tinham como foco questões relacionadas a: percepção da utilização das medidas de proteção individual pelos trabalhadores da instituição; dificuldades enfrentadas e estratégias utilizadas para melhorar a proteção no ambiente de trabalho; percepção da atuação da instituição com relação à problemática. O questionário foi incorporado ao protocolo de pesquisa quantitativa; porém, o seu preenchimento não era obrigatório.

### Análise e Tratamento dos Dados

Os dados quantitativos foram organizados em uma planilha eletrônica sob a forma de banco de dados no programa Excel/Windows e foram analisados no IBM-SPSS versão 25. A análise de dados foi realizada por meio da estatística descritiva (frequência absoluta e relativa) e inferencial (teste de Mann-Whitney ou Kruskal-Wallis). O teste de normalidade de Shapiro-Wilk mostrou que as variáveis não apresentaram distribuição normal. As escalas do Instrumento de Variáveis Relativas às PP foram representadas pela mediana e intervalo interquartílico tendo em vista a distribuição dos dados. O nível de significância de 5% (p < 0,05) foi adotado para todas as análises.

Os dados qualitativos foram submetidos à análise de conteúdo^(9)^ com auxílio do software MAXQDA. Após a organização do conjunto de dados a ser analisado (1ª etapa), realizou-se a exploração e análise aprofundada do material, com codificação e agrupamento (2ª etapa). Nessa etapa, os dados foram agrupados em uma categoria com 13 subcategorias. Por último, foi realizado o tratamento dos resultados e interpretação (3ª etapa).

Após, foi realizada a incorporação dos dados qualitativos aos quantitativos a partir da comparação dos achados, de forma que os itens da categoria qualitativa foram incorporados a resultados quantitativos. Essa integração foi realizada na etapa de interpretação dos resultados, buscando-se a complementaridade das informações e o aprofundamento na compreensão do objeto de estudo.

### Aspectos Éticos

Esta pesquisa foi submetida para consideração ao Comitê de Ética em Pesquisa das cinco instituições participantes e foi aprovada sob os seguintes pareceres: nº 4.335.006, nº 4.466.661, nº 4.685.755, nº 4.348.898 e nº 4.501.805. Todos os princípios éticos previstos em pesquisas envolvendo seres humanos foram respeitados de acordo com a Resolução 466/12. A participação na pesquisa foi precedida pela concordância online do participante, que teve acesso ao Termo de Consentimento Livre e Esclarecido. Além disso, a fim de preservar seu anonimato, as respostas qualitativas foram identificadas por códigos compostos pela letra “E” para enfermeiros, “M” para médicos, “TE” para técnicos de enfermagem, “AE” para auxiliares de enfermagem, e “G” para gestores e profissionais dos serviços de controle de infecção, saúde do trabalhador e educação permanente, seguidas de números associados à ordem em que o participante foi integrado à pesquisa. As instituições também foram identificadas por letras: Hospital A, Hospital B, e assim, sucessivamente.

## RESULTADOS

Na análise quantitativa, houve a participação de 559 profissionais, sendo 71% (n = 397) profissionais da enfermagem (195 técnicos de enfermagem, 185 enfermeiros, 17 auxiliares de enfermagem). Nessa amostra, 77,3% (n = 432) eram do sexo feminino, com média de idade de 45 (±9,54) anos (mínimo 22 e máximo 68 anos); 79,4% (n = 444) tinham companheiro e 72,8% (n = 407) possuíam filhos. Com relação às variáveis profissionais, 35,4% (n = 198) possuíam título de Especialista e 72,4% (n = 405) tinham seu contrato laboral regido pela Consolidação das Leis do Trabalho (CLT).

O tempo médio de experiência profissional foi de 18,3 anos (dp ± 9,1), o turno predominante de trabalho na instituição era o diurno para 67,1% (n = 375). Maior percentual (77,6%, n = 434) informou possuir um vínculo empregatício e 82,3% (n = 460) tinham uma carga de trabalho semanal ≤ 40 horas.

A Tabela 1 mostra os dados da associação entre as características sociodemográficas e ocupacionais e a adesão às PP.

**Tabela 1:** Comparação da distribuição da adesão às precauções padrão entre as variáveis categóricas sociodemográficas e ocupacionais (n = 559). Curitiba – Paraná; Florianópolis – Santa Catarina; Porto Alegre, Santa Maria e Rio Grande – Rio Grande do Sul, Brasil, 2020-2021.

n (%)	Adesão às PP med [P25; P75]	p-valor
**Sexo**		
Feminino 432 (77,3)	4,69 [4,46; 4,85]	0,440*
Masculino 127 (22,7)	4,62 [4,31; 4,85]	
**Situação conjugal**		
Com companheiro 444 (79,4)	4,69 [4,46; 4,85]	0,943*
Sem companheiro 115 (20,2)	4,69 [4,38; 4,85]	
**Filhos**		
Sim 407 (72,8)	4,69 [4,46; 4,85]	0,014*
Não 152 (27,2)	4,62 [4,38; 4,85]	
**Setor**		
Área COVID 53 (9,5)	4,77 [4,69; 4,92]	<0,001*
Área não-COVID 506 (90,5)	4,69 [4,38; 4,85]	
**Categoria profissional**		
Enfermagem 397 (71)	4,69 [4,46; 4,85]	0,630*
Médicos 162 (29)	4,69 [4,38; 4,85]	
**Vínculo de trabalho**		
RJU 127 (22,7)	4,69 [4,46; 4,85]	0,132**
CLT 405 (72,5)	4,69 [4,38; 4,85]	
Emergencial 27 (4,8)	4,77 [4,62; 4,92]	
**Formação (concluída)**		
Ensino médio 118 (21,1)	4,69 [4,54; 4,85]	0,347**
Graduação 71 (12,7)	4,69 [4,38; 4,92]	
Espec./residência 198 (35,4)	4,69 [4,38; 4,85]	
Mestrado 117 (21,0)	4,62 [4,38; 4,77]	
Doutorado 54 (9,7)	4,69 [4,46; 4,92]	
**Turno de trabalho predominante**		
Diurno 375 (67,1)	4,69 [4,38; 4,85]	0,582*
Noturno 183 (32,7)	4,69 [4,46; 4,85]	
**Outro vínculo empregatício**		
Não 434 (77,6)	4,69 [4,46; 4,85]	0,999*
Sim 125 (22,4)	4,69 [4,38; 4,85]	
**Carga horária semanal**		
≤40h 460 (82,3)	4,69 [4,46; 4,85]	0,921*
>40h 99 (17,7)	4,69 [4,38; 4,85]	

*Teste de Mann-Whitney; **Teste de Kruskal-Wallis. PP: precaução padrão; RJU: Regime Jurídico Ùnico; CLT: Consolidação das Leis do Trabalho.

As medianas das escalas do Instrumento de Variáveis Relativas às PP estão descritas na Tabela 2. Verificou-se nível alto para “adesão às PP” e níveis intermediários para as demais variáveis.

**Tabela 2:** Mediana e intervalo interquartílico das variáveis relativas às precauções padrão (n = 559). Curitiba – Paraná; Florianópolis – Santa Catarina; Porto Alegre, Santa Maria e Rio Grande – Rio Grande do Sul, Brasil, 2020-2021.

Escala	med [P25; P75]
Adesão às PP	4,69 [4,38; 4,85]
Eficácia da prevenção	4,00 [3,66; 4,67]
Percepção de risco	4,00 [3,33; 4,67]
Obstáculos para seguir as PP	4,00 [3,50; 4,67]
Carga de trabalho	4,00 [3,67; 4,67]
Clima de segurança	3,83 [3,25; 4,33]
Disponibilidade de EPI	4,00 [3,50; 5,00]

PP: precaução padrão; EPI: equipamento de proteção individual.

As variáveis relacionadas à pandemia que foram associadas a maiores valores de adesão às PP foram descritas na Tabela 3. Os profissionais que receberam orientações e/ou capacitação na instituição sobre biossegurança com foco na prevenção da transmissão do coronavírus tiveram valores significativamente maiores de adesão às PP. Aqueles que afirmaram que sempre realizaram o distanciamento social recomendado pela Organização Mundial da Saúde (OMS) nas demais atividades da vida particular também tiveram valores significativamente maiores.

**Tabela 3:** Comparação das medianas de adesão às precauções padrão com as variáveis relacionadas à pandemia de COVID-19 (n = 559). Curitiba – Paraná; Florianópolis – Santa Catarina; Porto Alegre, Santa Maria e Rio Grande – Rio Grande do Sul, Brasil, 2020-2021.

n (%)	Adesão às PP med [P25; P75]	p-valor
**Orientações e/ou capacitação na instituição sobre biossegurança com foco na prevenção da transmissão do novo coronavírus**		
Sim 523 (93,6)	4,69 [4,46; 4,85]	0,018*
Não 36 (6,4)	4,50 [4,15; 4,77]	
**Realizou o distanciamento social recomendado pela OMS nas demais atividades da sua vida particular?**		
Sempre 277 (49,6)	4,69a [4,54; 4,92]	<0,001**
Na maior parte do tempo 259 (46,3)	4,62b [4,38; 4,85]	
Ocasionalmente 13 (2,3)	4,46ab [4,23; 4,69]	
Raramente 10 (1,8)	4,58ab [4,00; 4,85]	

*Teste de Mann-Whitney; **Teste de Kruskal-Wallis; Post-hoc de Dunn: letras distintas representam distribuições estatisticamente diferentes. PP: precaução padrão; OMS: Organização Mundial de Saúde.

As associações significativas entre as Variáveis relativas às PP e as variáveis relacionadas à pandemia são descritas na Tabela 4. A mediana da percepção de risco foi mais elevada no grupo de participantes que teve sintomas sugestivos de COVID-19 durante o período. O grupo que afirmou ter recebido orientações/capacitação sobre biossegurança com foco na prevenção da transmissão do novo coronavírus teve melhor percepção quanto ao clima de segurança e melhor percepção quanto à disponibilidade de EPI. O grupo que afirmou sempre ter utilizado EPI durante a assistência direta a pacientes suspeitos ou confirmados de infecção por COVID-19 teve melhor percepção de clima de segurança e da disponibilidade de EPI.

**Tabela 4:** Comparação das medianas do Instrumento de Variáveis Relativas às Precauções Padrão com as variáveis relacionadas à pandemia de COVID-19 (n = 559). Curitiba – Paraná; Florianópolis – Santa Catarina; Porto Alegre, Santa Maria e Rio Grande – Rio Grande do Sul, Brasil, 2020-2021.

n (%)	Percepção de risco med [P25; P75]	p-valor	Clima de segurança med [P25; P75]	p-valor	Disponibilidade de EPI med [P25; P75]	p-valor
**Sintomas sugestivos de COVID-19**						
Sim 224 (40,1)	4,33 [3,67; 4,67]	< 0,001*	3,83 [3,17; 4,25]	0,218*	4,00 [3,50; 5,00]	0,118*
Não 335 (59,9)	4,00 [3,33; 4,33]		3,83 [3,33; 4,33]		4,50 [4,00; 5,00]	
**Orientações e/ou capacitação na instituição sobre biossegurança com foco na prevenção da transmissão do novo coronavírus**						
Sim 523 (93,6)	4,00 [3,33; 4,67]	0,812*	3,92 [3,33; 4,33]	< 0,001*	4,50 [4,00; 5,00]	0,001*
Não 36 (6,4)	4,00 [3,33; 4,67]		3,21 [2,67; 3,50]		3,50 [2,75; 4,50]	
**Uso de EPI durante o período de prestação de assistência e/ou realização de procedimentos a um paciente suspeito ou confirmado para COVID-19**						
Sempre 460 (82,3)	4,00 [3,33; 4,67]	0,169**	3,92a [3,33; 4,33]	0,001**	4,50a [4,00; 5,00]	0,001**
Na maior parte do tempo 92 (16,5)	4,00 [3,33; 4,67]		3,33b [2,96; 3,92]		3,50b [2,50; 4,00]	
Ocasionalmente 4 (0,7)	3,50 [3,17; 4,00]		2,58ab[1,75; 3,54]		2,75b [1,75; 3,00]	
Raramente 3 (0,5)	4,67 [4,33; 5,00]		2,83a [2,67; 4,17]		3,50a [3,50; 4,00]	

*Teste de Mann-Whitney; **Teste de Kruskal-Wallis; Post-hoc de Dunn: letras distintas representam distribuições estatisticamente diferentes. EPI: equipamento de proteção individual.

Com relação à análise qualitativa do estudo, os achados foram agrupados em uma categoria intitulada “Repercussões da pandemia na adesão às PP”, a qual evidenciou o entendimento dos profissionais quanto às medidas de proteção, quanto à percepção dos riscos, às questões relacionadas ao ambiente de trabalho e às instituições durante esse período. A unidade de análise utilizada na construção da categoria foi “Fatores relacionados à adesão às PP”. Essa categoria foi apresentada em 13 subcategorias, as quais foram incorporadas aos resultados quantitativos. Foram extraídas declarações significativas para melhor representá-los, conforme descrito no *Joint display* exibido no Quadro 1.

**Quadro 1:** *Joint display* com integração dos resultados quantitativos e declarações dos participantes. Curitiba – Paraná; Florianópolis – Santa Catarina; Porto Alegre, Santa Maria e Rio Grande – Rio Grande do Sul, Brasil, 2020-2021.

Repercussões da pandemia na adesão às precauções padrão		
Subcategoria	Resultados quantitativos (n = 559)*	Resultados qualitativos (n = 546 + 53)**
Adesão às PP	Adesão às PP [med] = 4,69 67% dos participantes afirmaram seguir às PP com todos os pacientes seja qual for o diagnóstico.	*“A implementação de precauções padrão constituiu a principal medida de prevenção da transmissão de COVID-19 entre pacientes e profissionais de saúde e foi adotada no cuidado a todos os pacientes, minimizando a exposição a patógenos respiratórios, incluindo o COVID-19.”* G46, Hospital C – Chefia de enfermagem do Centro Cirúrgico *“Penso que a pandemia auxiliou na reflexão da importância da precaução padrão para dia a dia de trabalho.”* G6, Hospital D – Chefia da Unidade de Cirurgia e Centro de Material e Esterilização
Uso de EPI	Uso de avental: 66% usam sempre quando há possibilidade de sujar as roupas com sangue ou outras secreções. Uso de luvas: 88% usam sempre quando há possibilidade de contato com sangue ou outras secreções. Uso de óculos de proteção ou protetor facial: 50% usam sempre quando há possibilidade de respingar sangue ou outras secreções nos olhos. Uso de máscara: 89% usam sempre quando há possibilidade de respingar sangue ou outras secreções na boca.	*“Os trabalhadores, diante da pandemia, entenderam a necessidade do uso dos EPI para se protegerem. O óbvio da proteção não era levado a sério durante as atividades diárias, por parte de alguns, antes da pandemia”*. G32, Hospital B – Serviço de Saúde Ocupacional e Segurança no trabalho *“A adesão ao uso de EPI parte de uma boa conscientização. Que agora está sendo feita. Os profissionais de saúde finalmente estão usando EPI sem termos que implorar.”* G53, Hospital D – Unidade de Gestão de Riscos “*Aderi com mais consciência o uso dos EPI*.” TE48, Hospital A – UTI *“O positivo disso tudo foram e são aprendizados diários que vieram com a pandemia e o reforço de práticas já existentes, com uso mais consciente. Por exemplo, os EPI.”* E133, Hospital C – Psiquiatria
Higiene das mãos	75% sempre realizam lavagem das mãos após retirar luvas descartáveis	*“Os profissionais aprenderam a valorizar as precauções para se protegerem e também aos pacientes, procurando impedir surtos e, principalmente, aprenderam a usar EPI. Nunca se falou tanto no tema higiene de mãos.”* G13, Hospital A - Serviço de Controle de Infecção Hospitalar
Cuidados com materiais e ambiente	86% consideram contaminados todos os materiais que estiveram em contato com saliva de pacientes.	*“Meu ambiente de trabalho atende a pacientes COVID e não COVID, e é muito importante que o ambiente não seja contaminado. Ficamos de olho em todos que saem da sala e não cuidam o ambiente.”* E123, Hospital C – Hemodinâmica
A família do profissional de saúde	Associação significativa entre adesão às PP e “ter filhos” (p = 0,014).	*“A assustadora possibilidade de contrair e transmitir à outras pessoas, sejam pacientes, familiares e amigos, moldou muitas atitudes. As práticas diárias com relação ao uso de EPI foram amplamente seguidas.”* E156, Hospital A – UNIDADE COVID
O trabalho em área COVID	Adesão às PP maior na área COVID (p < 0,001). Profissionais que trabalhavam em “área COVID” tiveram uma percepção de menos “obstáculos para seguir as PP” e uma melhor avaliação do “clima de segurança” e da “disponibilidade de EPI”.	*“É um local de maior probabilidade de contrair a doença, mas mais seguro na questão de proteção, pois nos protegemos mais com o uso dos EPI”.* TE15, Hospital A – UNIDADE COVID “*Na área COVID, os funcionários se paramentam adequadamente. No entanto, na área não COVID, há algum relaxamento no uso das medidas de precaução*.” G28, Hospital C – Serviço de Educação em Enfermagem *“... a adesão dentro da UTI COVID é visivelmente maior pensando no viés de proteção do profissional.”* G9 – Hospital D – Chefia da Unidade de Terapia Intensiva
Percepção da eficácia da proteção	86% dos profissionais acreditam que podem diminuir o risco de adquirir a COVID-19 no trabalho se seguirem às precauções padrão.	*“Acredito que os profissionais de saúde usando tudo corretamente têm um risco diminuído de adquirir o vírus no trabalho, a gente se cuida mais com certeza.”* M92, Hospital B – Pediatria
Percepção do risco	6% dos participantes não se sentem expostos a contrair COVID-19 no trabalho. A “percepção de risco” é maior entre profissionais da enfermagem (p < 0,001) e no grupo de participantes que teve sintomas sugestivos de COVID-19(p < 0,001).	*“A pandemia provocou medo nos profissionais, o que levou muitos a utilizarem os EPI de forma adequada. No entanto, existem momentos de negação, nos quais os profissionais ignoram as medidas de proteção indicadas.”* G27, Hospital C – Chefia do Serviço de Enfermagem Psiquiátrica *“As dificuldades são de entender porque algumas pessoas não querem aceitar que o vírus é letal, e, muitas vezes, não usam normas de proteção.”* TE111, Hospital B – Ambulatório
Obstáculos para adesão às PP	24% afirmaram que com frequência o acúmulo das atividades diárias interfere na capacidade de seguir as PP. Cerca de 20% afirmaram que não conseguem se acostumar com o uso de EPI na realização de algumas tarefas e acreditam que seguir as PP torna o trabalho mais difícil.	*“Face shield distorce a visão e atrapalha para intubação traqueal.”* M47, Hospital C – Centro cirúrgico *“Pressão por agilidade no atendimento que nem sempre é possível tendo protocolos de paramentação para seguir.”* TE48, Hospital A – UTI *“Dificuldade, às vezes, em me equipar rapidamente quando surge emergência que exige rapidez.”* M118, Hospital D – Pronto-Socorro *“Muito difícil nos ouvirmos com máscara e protetor facial. Temos de gritar e isso torna o trabalho muito mais desgastante.”* M48, Hospital C – UTI
Carga de trabalho	35% dos participantes afirmaram que sempre existe muito trabalho a ser feito. A categoria de médicos teve uma maior percepção de “carga de trabalho” comparado à enfermagem (p = 0,002).	*“Em relação especificamente ao hospital, tendo que realizar atividades que em outras instituições são atribuições da enfermagem (como coleta de swab nasofaringe, ECG, aferição de sinais vitais dos pacientes com sintomas respiratórios), o que torna o trabalho ainda mais exaustivo.”* M4, Hospital D – Pronto Socorro
Clima de segurança	Clima de segurança foi a escala com escore mais baixo (Md = 3,83). 68% concordam totalmente que tem apoio do supervisor para seguir as PP. 16% concordam totalmente que na sua instituição a alta gerência se envolve pessoalmente nas atividades de segurança. Hospital C teve a melhor avaliação do “clima de segurança” (4,17) e o Hospital D, a pior (3,25) (p < 0,001). Médicos (p = 0,009) e profissionais que são regidos pela CLT (p < 0,001) tiveram uma melhor avaliação desse item.	*“Um pouco falha por parte de gestores que NÃO atuam na assistência e têm uma percepção um pouco distorcida das rotinas de trabalho.”* TE80, Hospital D – UTI “*Percebo que a instituição apenas quer ‘mão de obra’, não está verdadeiramente preocupada com a saúde do colaborador (física e mental)*.” TE20, Hospital D – UNIDADE COVID *“Sinto o Hospital bastante engajado na segurança de todos os seus profissionais, atende a todas as nossas necessidades protetivas.”* E102, Hospital C - Enfermaria *“Desde o início da pandemia, fomos todos orientados pela chefia. E isso nos deixou muito seguros para lidar com todas as situações frente à pandemia.”* TE84, Hospital C - UTI *“Eficaz, orientações corretas. Os gestores participaram junto à equipe, questionando quais as melhores evidências para a segurança da equipe.”* TE146, Hospital C – UNIDADE COVID *“No início da pandemia a instituição focava o apoio muito nas unidades de tratamento intensivo, como se fosse o único meio de transmissão, senti insegurança. Após, com o surgimento de casos e contaminação nas unidades, os processos foram melhorando.”* E133, Hospital C – Psiquiatria
Disponibilidade de EPI	48% de todos os participantes afirmaram que a sua unidade de trabalho possui todos os equipamentos e materiais necessários para proteção da exposição à COVID-19. Médicos (p = 0,04) tiveram uma melhor percepção de “disponibilidade de EPI”.	“...*no início da pandemia, não dispunha de EPI em número correto para toda a equipe, sendo dispensado máscara N95 inicialmente apenas para equipe médica, de fisioterapia e enfermeiros, não sendo dispensada para técnicos de enfermagem, o que provocou desconforto*...” E2, Hospital A – Centro Cirúrgico *“Acho que foi feito o possível, às vezes temos EPI de qualidade questionável, como os aventais descartáveis, mas nunca faltou material, o que considero um feito a ser destacado.”* M91, Hospital B – UTI
Treinamento/ capacitação em biossegurança	Ter recebido orientações/capacitação sobre biossegurança com foco na prevenção da transmissão ao novo coronavírus foi associado a maiores valores de adesão às PP (p = 0,018).	*“Houve uma preocupação maior para aquisição de conhecimento e busca por material adequado.”* G11, Hospital D – Serviço de Controle de Infecção Hospitalar *“O que temos de maneira positiva são ‘lives’ esclarecedoras com relação ao assunto.”* E16, Hospital A – Ambulatório

*n = 559: profissionais da enfermagem e médicos que atuavam na assistência. **n = 543 +53: profissionais da enfermagem e médicos que atuavam na assistência + gestores dos serviços de saúde e profissionais que atuavam nos serviços de controle de infecção, saúde do trabalhador e serviço de educação permanente.

## DISCUSSÃO

Os resultados permitiram analisar a adesão às PP, por profissionais da saúde, durante a pandemia. Estudos anteriores apresentaram níveis intermediários de adesão às PP^(10–13)^, diferentemente do nível elevado encontrado no presente estudo. As respostas qualitativas corroboram esse achado, demonstrando que a pandemia levou a uma maior conscientização quanto à utilização das PP.

O medo de contrair e de transmitir a doença aos familiares foi identificado como um aspecto que influenciou na adesão às PP, sendo que os profissionais que possuem filhos foram mais aderentes. O contexto da pandemia fez com que os profissionais refletissem sobre os riscos a que estão expostos no ambiente de trabalho, gerando mudanças nas atitudes e comportamentos, o que resultou em uma maior adesão às PP. A literatura aponta que o medo de se infectar ou infectar familiares está entre os principais desafios mentais enfrentados por profissionais da saúde que atuam em pandemias, o que, somado ao estigma sofrido por parte da sociedade, gera estresse e isolamento^(14)^. Assim, aderir às PP, além do benefício relacionado à proteção física, contribui para o bem-estar emocional do trabalhador, que passa a se sentir seguro para realizar sua atividade.

Os profissionais mais aderentes às PP eram os que atuavam nas unidades destinadas ao tratamento exclusivo de pacientes com COVID-19. Segundo os resultados qualitativos, os profissionais dessas unidades tiveram menor dificuldade para seguir as PP e receberam mais apoio institucional, tanto no que diz respeito à disponibilidade de equipamentos quanto à participação ativa dos gestores junto às equipes. Somado a isso, acredita-se que o elevado risco de contaminação nesses locais foi um fator que contribuiu positivamente na motivação para que os profissionais aderissem às práticas de segurança com rigor.

Um clima de segurança elevado em unidades de atendimento à COVID-19 foi observado em outros países. Na Espanha, os profissionais dessas áreas sentiram-se privilegiados por estarem suficientemente equipados e não foram observadas altas taxas de contágio entre eles^(15)^. Outros estudos demonstraram que a contaminação de profissionais da saúde foi maior em enfermarias, fato que os pesquisadores atribuem a uma diferença no uso de EPI e nas práticas de biossegurança entre os locais para atendimento exclusivo a pacientes com COVID-19 e locais de atendimento geral^(16,17)^.

No presente estudo, os achados demonstraram que os profissionais mais aderentes ao uso de EPI, durante assistência ao paciente com COVID-19, tiveram uma melhor percepção quanto ao clima de segurança e disponibilidade de EPI, o que se considera uma evidência de que se sentir apoiado é um fator relevante para estimular a adesão às PP.

É importante destacar que o clima de segurança percebido pelos participantes teve uma diferença significativa entre as instituições. Na instituição que teve a melhor avaliação dessa escala, Hospital C, os participantes relataram uma participação ativa das chefias junto às equipes, com orientações claras e eficazes, e buscando as melhores evidências para a segurança de todos. Isso fez com que os profissionais percebessem o engajamento da instituição na segurança dos profissionais, buscando atender às suas necessidades de proteção, o que melhorou a percepção quanto ao clima de segurança.

Nesse sentido, o item avaliado pior pelos participantes do estudo, no que diz respeito ao clima de segurança, foi o envolvimento da alta gerência nas atividades de segurança. Estudo com profissionais de enfermagem brasileiros apontou que a maioria (81,8%) deles não se sentia seguro com as ações implementadas pelas instituições no enfrentamento da COVID-19^(18)^. O sentimento de apoio inadequado, somado a uma alta carga de trabalho e à falta de EPI, repercutiram na carga mental dos profissionais^(19)^. Os profissionais precisam se sentir apoiados por suas instituições e por seus líderes e, assim, estimulados a assumir a sua própria responsabilidade para garantir um ambiente de trabalho mais seguro.

Os profissionais da saúde demonstraram elevado interesse em aprender sobre as medidas de biossegurança. Além disso, as instituições de saúde ofertaram diversos tipos de treinamentos, capacitações e orientações sobre o tema, o que favoreceu a disseminação do conhecimento relacionado às PP. A variável “Ter recebido orientações e treinamento sobre biossegurança” foi associada estatisticamente a uma melhor percepção do clima de segurança e disponibilidade de EPI. Também, os profissionais mais aderentes às PP foram os que receberam treinamento na própria instituição.

Com relação à disponibilidade de EPI, identificou-se que um percentual de 48% afirmou que suas unidades de trabalho possuíam todos os equipamentos necessários para sua proteção. Na análise qualitativa, identificou-se que as unidades destinadas ao atendimento de paciente com COVID-19 foram mais bem contempladas quanto à oferta desses equipamentos, o que também foi demonstrado na literatura^(15)^. Um estudo realizado na UTI de um hospital universitário do Canadá verificou que a disponibilidade de EPI foi uma preocupação constante durante a pandemia. A angústia dos profissionais estava relacionada à possibilidade de que não haveria equipamentos suficientes, somada à necessidade de utilização de produtos desconhecidos até então^(20)^. Sobre isso, é importante enfatizar que ter condições para realização de um trabalho seguro, especialmente em um momento de crise sanitária, é relevante para a saúde física e mental dos profissionais da saúde.

A adesão às PP foi associada ao cumprimento da recomendação de distanciamento social. Os indivíduos que mais seguiram as normativas de segurança no ambiente de trabalho demonstraram que estenderam esses cuidados ao ambiente extra-hospitalar, sinalizando para uma coerência de atitudes na busca por uma proteção maior com relação à infecção pelo vírus. Essas ações realizadas em conjunto são essenciais, tendo em vista que a eficácia do distanciamento social depende em grande parte da adoção das demais medidas, como correta higienização das mãos, uso de máscara e medidas de higiene de superfícies^(21)^.

Perceber o risco a que se está exposto é considerado um fator importante para a atitude dos profissionais da saúde com relação à utilização das medidas de proteção. Os achados do presente estudo demonstraram que os profissionais com sintomas para COVID-19 tiveram uma maior percepção de risco para infecção por SARS-CoV-2. Esse aspecto é importante, pois a falta de consciência dos riscos contribui para o comportamento de desproteção. Estudo demonstrou que, apesar dos graves acidentes de trabalho envolvendo material biológico, existe uma subestimação dos riscos tanto por parte dos empregadores como dos empregados^(22)^. Durante a pandemia, a exposição a um risco até então desconhecido aumentou essa consciência dos profissionais, influenciando a adoção das medidas protetivas. Entretanto, é preciso considerar que a convivência permanente com a exposição favorece a diminuição da percepção dos riscos, dificultando a decisão pela adoção das PP^(22)^. Assim, novos estudos e intervenções terão de ser desenvolvidos nos anos conseguintes para melhor avaliar as mudanças com relação à percepção de risco e a sua associação com a adoção de medidas de segurança pelos profissionais da saúde.

Os resultados também evidenciaram elevada adesão ao uso de EPI, como avental, máscara, luvas e óculos de proteção. Esse resultado é positivo quando comparado a um estudo anterior realizado apenas com profissionais da enfermagem^(12)^. Tanto os resultados quantitativos como os qualitativos evidenciaram que os participantes acreditavam ser possível atender pacientes infectados sem se contaminar, fazendo uso da proteção adequada. De fato, estudos demonstram que a observância das indicações de uso dos EPIs é eficiente para prevenir as infecções entre os profissionais da saúde^(23)^. Na China, pesquisadores buscaram examinar os efeitos de proteção de EPI para profissionais que prestavam atendimento a pacientes com COVID-19 e demonstraram que, apesar do alto risco de exposição, nenhum dos 420 profissionais foi infectado, mesmo realizando procedimentos geradores de aerossol, pois estavam adequadamente protegidos^(24)^.

Apesar do benefício da proteção, a utilização de EPI tem implicações negativas para a realização do trabalho. Cerca de 20% afirmaram que não conseguem se acostumar com a utilização do EPI e acreditavam que seguir as PP torna o trabalho mais difícil. A dificuldade para realizar as tarefas com os EPIs envolve a diminuição dos sentidos, como visão, audição e tato, interferindo na capacidade de realizar o trabalho de forma eficiente. Além disso, o desconforto físico gerado pela utilização do EPI tornou a adesão por períodos prolongados um desafio a ser superado. Uma pesquisa com enfermeiros apontou como desconfortos mais comuns relacionados ao uso de EPI: sudorese ao usar máscara cirúrgica (50,9%) ou do tipo N95 (64,2%), ressecamento das mãos pela constante lavagem e uso de luvas (73,9%), transpiração pelo uso de macacão/avental (84,1%) e problemas de visão e cefaleia ao utilizar óculos de proteção/protetor facial (47,9%)^(25)^. Esses desconfortos precisam ser considerados quando se pensa em aumentar a adesão, indicando a necessidade de mais pesquisas que examinem a qualidade, a eficácia e o conforto dos EPIs.

Além do uso de EPI, o aumento na higienização das mãos foi uma das mudanças relatadas pelos participantes. Entretanto, os resultados quantitativos apontaram um percentual de 75% dos profissionais que sempre realizam a lavagem das mãos após a retirada de luvas. Estudos realizados antes da pandemia demonstraram uma maior prática de higiene das mãos, chegando a 83,6% entre profissionais da enfermagem de um hospital universitário^(12)^ e 82,9% em outro estudo realizado em hospital psiquiátrico^(11)^.

Estudo realizado em Cingapura verificou a contaminação do ambiente em áreas de isolamento para pacientes positivos para COVID-19 em distâncias maiores de um metro, demonstrando que a contaminação provavelmente foi ocasionada pelas mãos dos profissionais da saúde^(26)^. Em uma amostra com 72 profissionais da saúde em Wuhan, na China, pesquisadores evidenciaram que a lavagem de mãos não qualificada, a higiene subótima antes do contato com o paciente e o uso inadequado de EPI foram fatores de risco para infecção por SARS-CoV-2 nos trabalhadores^(27)^. Dessa forma, está claro o papel central que a adesão às PP tem tanto na proteção dos profissionais da saúde quanto na segurança dos pacientes internados, amparando a preocupação para que os profissionais deixem de ser vetores de infecção no ambiente hospitalar por meio da aplicação rigorosa das medidas de biossegurança.

Mesmo que os resultados deste estudo tenham encontrado respaldo na literatura, algumas limitações podem ser apontadas. A utilização de instrumento autoaplicável, que está sujeito ao viés de memória do participante, pode interferir nos resultados obtidos. O delineamento transversal utilizado impossibilita o estabelecimento de relações de causa e efeito. Por fim, reconhecemos a característica não probabilística da amostra, a qual obteve mais participantes da categoria da enfermagem, mesmo que o estudo tenha sido divulgado igualmente para os profissionais médicos das instituições participantes.

Os achados deste estudo poderão nortear novas intervenções no processo de educação nas instituições, buscando tanto a proteção dos profissionais quanto a dos pacientes. As estratégias devem estar focadas em mudanças consistentes no comportamento de risco dos profissionais, além de melhoria das condições de trabalho, que envolve dimensionamento adequado de pessoal e provisão de material e EPI suficientes e de qualidade para todos os profissionais que prestam assistência direta ou que estão expostos aos riscos relacionados a ela. O apoio ofertado pelas instituições deve contar com a participação de toda a equipe nas atividades de segurança, do nível assistencial ao gerencial.

Recomenda-se o monitoramento da adesão às práticas de segurança, com manutenção de estratégias educativas, e a criação de programas institucionais de atenção à saúde do trabalhador.

## CONCLUSÃO

O estudo identificou alto nível de adesão às PP por profissionais da saúde durante a pandemia de COVID-19. As variáveis relativas às PP obtiveram níveis intermediários. Os fatores que foram associados a maior adesão às PP foram: ter filhos, trabalhar em unidades de atendimento à COVID-19, receber orientações/capacitação na instituição sobre biossegurança e realizar distanciamento social recomendado pela OMS. Também contribuíram de forma positiva para a adesão às PP nesse período a percepção de risco elevada dos profissionais da saúde, uma expressiva busca pela utilização de equipamentos de proteção e a propagação de conhecimentos relacionados à biossegurança.
